# Next generation of antiretroviral agents targeting the RNA binding site of the HIV-1 cellular cofactor DDX3: an innovative therapeutic approach

**DOI:** 10.1186/1742-4690-9-S1-P5

**Published:** 2012-05-25

**Authors:** Giovanni Maga, Anna Garbelli, Marco Radi, Federico Falchi, Alberta Samuele, Stefania Paolucci, Fausto Baldanti, Fabrizio Manetti, Sandra Beermann, Ursula Dietrich, Maurizio Botta

**Affiliations:** 1 Instituto di Genetica Molecolare, IGM-CNR Via Abbiategrasso 207, I-27100 Pavia, Italy; 2 Dipartimento Farmaco Chimico Tecnologico, University of Siena,Via Alcide de Gasperi 2, I-53100 Siena, Italy; 3 Molecular Virology Unit, Foundation IRCCS Policlinico S. Matteo, piazzale Golgi, I-27100 Pavia, Italy; 4 Georg-Speyer-Haus Institute of Biomedical Research, Paul-Ehrlich-Str. 42-44, 60596 Frankfurt, Germany

## Introduction

Efficacy of currently approved anti-HIV drugs is hampered by mutations of the viral enzymes, leading invariably to drug resistance and chemotherapy failure. Recent data suggest that cellular co-factors also represent useful targets for anti-HIV therapy. We have recently provided evidence for the possibility to block HIV-1 replication by targeting its cellular cofactor DDX3.

## Material and methods

Molecular modeling and in silico technologies were applied to rationally design small molecules specifically targeting the RNA binding site of human DDX3. Biochemical studies of mutated DDX3 enzymes were also used to identify additional potential drug binding sites.

## Results

Optimization of compounds identified by application of a high-throughput docking approach afforded a promising lead compound which proved to inhibit both the helicase and ATPase activity of DDX3 and to reduce the viral load of peripheral blood mononuclear cells (PBMC) infected with HIV-1. A novel interaction site has been also identified in DDX3, which, when blocked, can reduce viral replication, representing an additional target for small molecules inhibitors.

## Conclusions

We have identified the first inhibitors of HIV-1 replication targeting the RNA binding site of the cellular cofactor human DDX3. These compounds may offer superior selectivity over the ATP-competitive inhibitors previously developed. In addition, a novel RNA interacting motif specific to DDX3 has been identified, opening new venues for HIV-1 drug development.

